# Application of Near-Infrared Spectroscopy and Fuzzy Improved Null Linear Discriminant Analysis for Rapid Discrimination of Milk Brands

**DOI:** 10.3390/foods12213929

**Published:** 2023-10-26

**Authors:** Xiaohong Wu, Yiheng Fang, Bin Wu, Man Liu

**Affiliations:** 1 School of Electrical and Information Engineering, Jiangsu University, Zhenjiang 212013, China; wxh419@ujs.edu.cn (X.W.); 17857578515@163.com (Y.F.); 18186214786@163.com (M.L.); 2 High-Tech Key Laboratory of Agricultural Equipment and Intelligence of Jiangsu Province, Jiangsu University, Zhenjiang 212013, China; 3 Department of Information Engineering, Chuzhou Polytechnic, Chuzhou 239000, China

**Keywords:** milk, near-infrared spectroscopy, improved null linear discriminant analysis, Savitzky–Golay filtering, K-nearest neighbor

## Abstract

The quality of milk is tightly linked to its brand. A famous brand of milk always has good quality. Therefore, this study seeks to design a new fuzzy feature extraction method, called fuzzy improved null linear discriminant analysis (FiNLDA), to cluster the spectra of collected milk for identifying milk brands. To elevate the classification accuracy, FiNLDA was applied to process the near-infrared (NIR) spectra of milk acquired by the portable near-infrared spectrometer. The principal component analysis and Savitzky–Golay (SG) filtering algorithm were employed to lower dimensionality and eliminate noise in this system, respectively. Thereafter, improved null linear discriminant analysis (iNLDA) and FiNLDA were applied to attain the discriminant information of the NIR spectra. At last, the K-nearest neighbor classifier was utilized for assessing the performance of the identification system. The results indicated that the maximum classification accuracies of LDA, iNLDA and FiNLDA were 74.7%, 88% and 94.67%, respectively. Accordingly, the portable NIR spectrometer in combination with FiNLDA can classify milk brands correctly and effectively.

## 1. Introduction

As an essential nutritional source for numerous people [1], milk encompasses multiple nutritional or non-nutritional elements, such as functional and bioactive substances, lipids [2], and proteins [3], as well as minerals [4], vital amino acids, and lactose [5]. Although the milk of all mammals possesses the same primary components, namely carbohydrates, water, minerals, proteins, vitamins, and fats, the milk of cud-chewing and non- cud-chewing animals varies substantially in the contents of these components [6]. The composition of milk is also affected by the frequency and stage of lactation, heredity, somatic cell count, animal diet, the treatment of milk, and seasonal variations [7,8,9,10,11].

However, milk is a major object of adulteration because of its enormous economic benefits. The nutritional assessment and adulteration detection of milk has become increasingly challenging due to the features of milk, such as special composition, the broad range of animal origins, and many influencing factors for nutrition. The evolution of food omics in recent years has enabled the increasing application of various techniques for rapidly screening or selectively confirming milk quality and authenticity.

Near-infrared (NIR) spectroscopy coupled with multivariate processing techniques has been extensively analyzed in recent years. This technology has been significantly applied in several industries, like the agricultural product industry [12,13], the pharmaceutical factory [14] and the petrochemical industry [15,16]. NIR has become a pivotal assessment method for milk quality [17,18] because it is an undamaged, green and rapid method. More importantly, NIR analysis is free of chemical reagents in line with the principle of green chemistry. In addition to these advantages, NIR spectroscopy has been validated to be utilized for the good analysis of food and agricultural products because NIR mainly records the frequency-doubled and frequency-combined absorption of the hydrogen-containing group X-H (X = C, N, O) vibration [19]. NIR can be used in identifying food varieties, and furthermore, the analytical methods require discriminant analysis and classifiers. The portable NIR instrument has multiple advantages, such as the ability of emergency analysis, a short test time, an undamaged sample, a small sample size, and easy maintenance, but the spectrum detection range is narrow [20].

Given the serious overlap of spectra in the NIR spectrum region, if NIR spectra are directly classified, the classification accuracy will be low. To improve the classification accuracy, NIR spectra should be processed by a feature extraction method before classification. Improved null linear discriminant analysis (iNLDA) is an improved feature extraction method based on null linear discriminant analysis (NLDA) [21]. iNLDA and NLDA have similar capability in most cases, but iNLDA further cuts down the computational cost of NLDA. Nevertheless, for the within-class scatter matrix constructed by the low-dimensional data, its null space is empty, and neither NLDA nor iNLDA is applicable [22]. In order to obtain a faster, more accurate and more flexible algorithm to process the NIR spectra of collected milk, this paper presented a novel fuzzy feature extraction method, called improved null linear discriminant analysis (FiNLDA). And a combination of portable NIR spectrometer and FiNLDA was designed to identify milk brands.

## 2. Materials and Methods

### 2.1. Sample Preparation

In this study, five brands (Guangming, Mengniu, Telunsu, Yili, Jindian) of milk samples came from the local supermarket in China. For the same brand of milk samples, they came from the same manufacturer, and their production batch and production date are the same; for different brands of milk samples, their production dates are close. There were 60 samples for each brand, totaling 300 samples. Thereafter, all milk samples were categorized into training and test samples according to a certain proportion. The milk samples met the following requirements: milk capacity (250 mL), packaging (carton/plastic shell) and in the shell life.

### 2.2. NIR Spectra Collection

**Figure 1:**
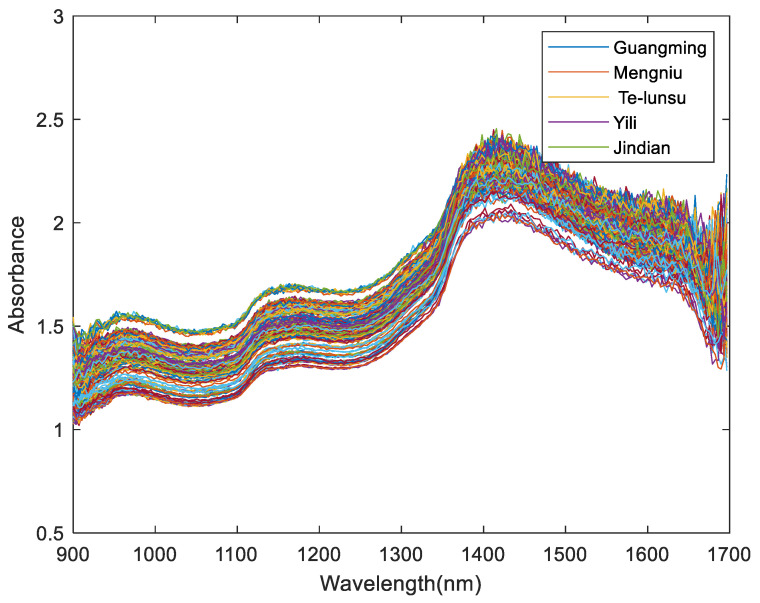
The raw spectra of milk.

The NIR spectral data of milk samples were acquired using the NIR-M-R2 spectrometer made by Shenzhen Pynect limited corporation, Shenzhen, China. The wavelength range of the spectrometer ranges from 900 nm to 1700 nm, and its resolution is 10 nm. The NIR data were collected at about 25 °C with relative humidity of 50–60% throughout the collection process. The spectrometer should be warmed up for one hour before collecting near-infrared spectral data. The collected near-infrared spectrum of milk is the 228-dimensional data. Each milk sample was scanned three times, and the final datum was the average of the three test results. The NIR spectra of milk samples are shown in Figure 1.

### 2.3. NIR Spectra Preprocessing

**Figure 2:**
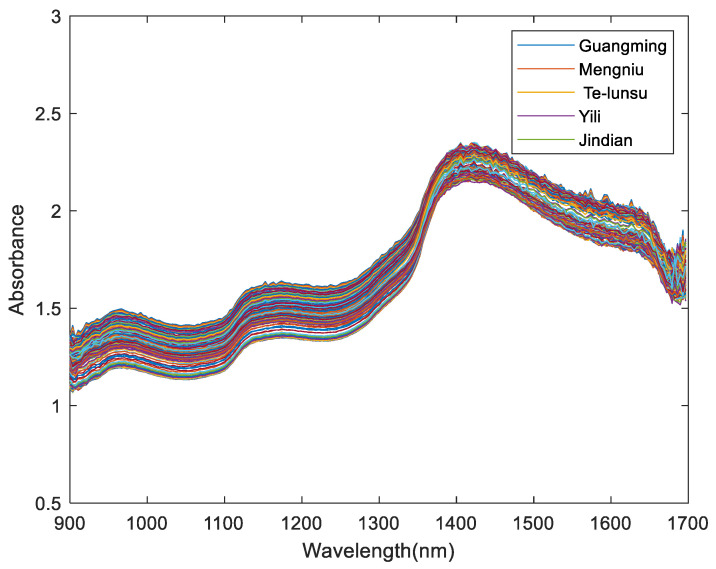
The preprocessed spectra of milk.

The original spectrum is greatly affected by physical conditions. The data shown in Figure 1 were mixed with a noise signal in addition to the sample characteristics [23]. In order to filter out the noise signal, the NIR spectra needed to be preprocessed, and the Savitzky–Golay (SG) filtering method was applied to preprocess the NIR spectra [24]. Regarding the SG filter, the sgolayfilt (X, order, frame) function in MATLAB was performed to preprocess the data. This experiment defined order as 1 and frame as 11. The functions of setting these parameters are to remove scattering, cut down the influence of diffuse reflection, reduce random errors, remove the redundant data, etc. [22,25]. After preprocessing, the NIR spectra are shown in Figure 2.

### 2.4. Improved Null Linear Discriminant Analysis

The procedure of iNLDA is described as follows [22]:

(1) Build matrices $H_{t}$, $H_{b}$, $H_{w}$ by the training data (containing *n* data points in ${\mathbb{R}}^{m}$);
(2) Performing singular value decomposition on the matrix $H_{t}$, $H_{t}=U_{1}\Sigma_{r}V_{1}$, $\Sigma_{r}\in {\mathbb{R}}^{r\times r}$, $U_{1}\in {\mathbb{R}}^{m\times r}$, $U_{2}\in {\mathbb{R}}^{m\times \left(n-r\right)}$. $r=rank(S_{t})$;
(3) Construct the matrix $\tilde{S_{w}}=U_{1}^{T}S_{w}U_{1}$;
(4) Perform the eigendecomposition of matrix $\tilde{S_{w}}$. The matrix W is constructed by the eigenvectors associated with the zero eigenvalues;
(5) Define the matrix $G=U_{1}W$. Here, G is the feature projection matrix of iNLDA.

### 2.5. Fuzzy Improved Null Linear Discriminant Analysis

The procedure of FiNLDA is described as follows (For the calculation of the initial fuzzy membership degree, see Formula (1) in the Ref. [26]):

(1) Build matrices $H_{ft}$, $H_{fb}$, $H_{fw}$ by the training data (containing *n* data points in ${\mathbb{R}}^{p}$:

(1) $$H_{ft}=\left[\sqrt{\sum_{j=1}^{c}u_{1j}^{m}}\left(x_{1}-\bar{x}\right),\;\sqrt{\sum_{j=1}^{c}u_{2j}^{m}}\left(x_{2}-\bar{x}\right),\ldots ,\;\sqrt{\sum_{j=1}^{c}u_{nj}^{m}}\left(x_{n}-\bar{x}\right)\right]$$

(2) $$H_{fb}=\left[\sqrt{{\left(U_{1}^{f}\right)}^{T}e}\left(v_{1}-\bar{x}\right),\;\sqrt{{\left(U_{2}^{f}\right)}^{T}e}\left(v_{2}-\bar{x}\right),\ldots ,\sqrt{{\left(U_{c}^{f}\right)}^{T}e}\left(v_{c}-\bar{x}\right)\right]$$

(3) $$H_{fw}=\left[A_{1},A_{2},\ldots ,A_{c}\right]$$

Here, $U_{j}^{f}$ and $A_{j}$ in (2) and (3), are defined as

(4) $$U_{j}^{f}={\left[u_{1j}^{m},\;u_{2j}^{m},\ldots ,\;u_{nj}^{m}\right]}^{T}$$

(5) $$A_{j}=\left[\sqrt{u_{1j}^{m}}(x_{1}-v_{j}),\;\sqrt{u_{2j}^{m}}(x_{2}-v_{j}),\ldots ,\;\sqrt{u_{nj}^{m}}(x_{n}-v_{j})\right]$$

where $\bar{x}$ is the mean of all samples $\bar{x}=\frac{1}{n}\sum_{i=1}^{n}x_{i}=\frac{1}{n}Xe$, $e={\left[1,\;1,\ldots ,\;1\right]}^{T}\in {ℜ}^{n}$.

(2) Performing singular value decomposition on the matrix $H_{ft}$, $H_{ft}=U_{1}\Sigma_{r}V_{1}$, $U_{1}\in {\mathbb{R}}^{p\times r}$,$U_{2}\in {\mathbb{R}}^{p\times \left(n-r\right)}$. $r=rank(S_{ft})$;
(3) Construct the matrix $\tilde{S_{fw}}=U_{1}^{T}S_{fw}U_{1}$;
(4) Perform the eigendecomposition of matrix $\tilde{S_{fw}}$. The matrix W is constructed by the eigenvectors associated with the zero eigenvalues;
(5) Define the matrix $G=U_{1}W$. Here, G is the feature projection matrix of FiNLDA.

### 2.6. K-Nearest Neighbor

One superiority of the K-nearest neighbor (KNN) method is its simple calculation, but it does not affect its classification effect. In some experiments, KNN can even achieve better classification results than other more complex classifiers. For KNN, the K-nearest samples closest to the unknown object are chosen and the majority rule is applied: the unknown object is categorized into the class to which most of the K samples belong to. The selection of K is performed by calculating the predictive power for different values of K, with small values of K (3 or 5) generally being preferred [27].

### 2.7. Software

In this experiment, MATLAB R2022a (The MathWorks, Co., Ltd., Portolla Valley, CA, USA) was utilized to deal with the data and calculate the classification accuracy.

## 3. Results

### 3.1. Dimensional Reduction by PCA

In this experiment, the NIR spectral data of our milk samples were adopted to construct a data matrix of 300 × 228, where 300 is the sample number and 228 is the sample dimension. Since the data matrix contains feature information irrelevant to the analysis, this experiment used principal component analysis (PCA) to reduce the dimension and redundancy of the data. After PCA processing, the reduced dimension matrix including original features, orthogonal features and integrated features can be obtained.

The number of principal components (PCs) can markedly impact the classification accuracy of the recognition system. Accordingly, it is highly critical for selecting the proper number of PCs to improve the classification accuracy [28].

**Figure 3:**
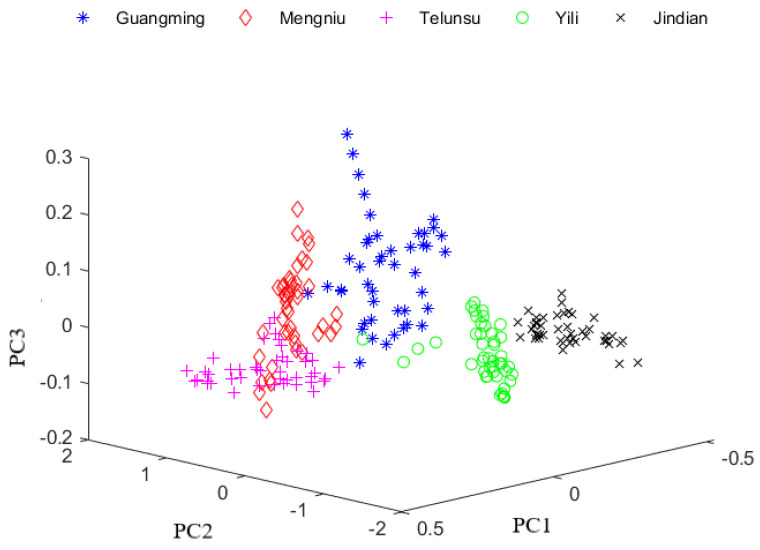
Data distribution of training set under S-G filter + PCA.

The number of principal components is usually selected based on the cumulative contribution rate. When the rate reaches 90%, it proves that the principal components contain most of the effective information. Since the first 20 principal components constitute 99% of the total variance, they retain the feature data of the near-infrared spectral data, and eliminate the redundant data. After 20 feature vectors were calculated by PCA, the 228-dimensional spectra were projected into these vectors to produce the 20-dimensional data. Furthermore, a three-dimensional feature space constructed by the first three principal components was established to observe the near-infrared spectral data of milk. The three-dimensional display of the training data after PCA processing is shown in Figure 3. After PCA, the 300 milk samples were divided into a training set (45 training samples per brand, 225 in total) and a test set (15 test samples per brand, 75 in total).

The classification of PCA + LDA, PCA + iNLDA and PCA + FiNLDA for milk brands were introduced in the following sections.

### 3.2. Discriminant Feature Extraction by LDA

**Figure 4:**
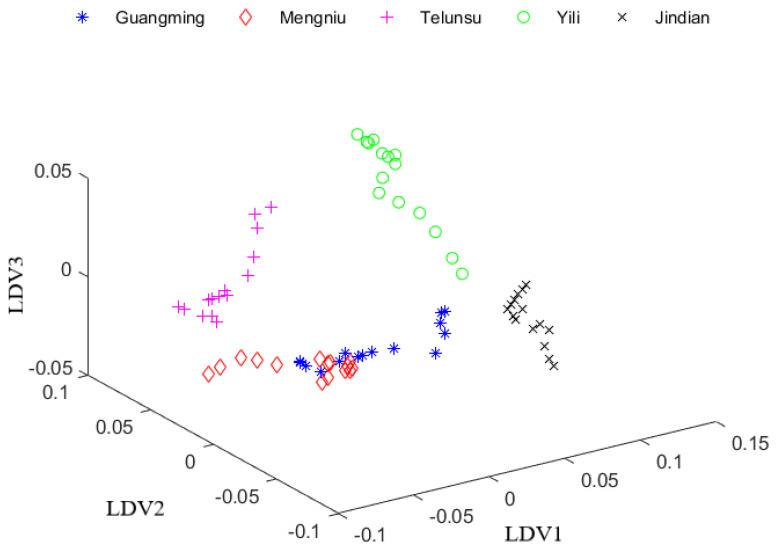
The data distribution of test set after PCA + LDA.

After PCA reduced the dimension of the NIR data to 20 dimensions, linear discriminant analysis (LDA) was applied to extract the discriminant features from the training set. For LDA, the number of eigenvectors and eigenvalues is usually the category number minus one. Therefore, the eigenvalues of four features were computed and listed: $\lambda_{1}\;$= 113.2, $\lambda_{2}\;$= 51.6, $\lambda_{3}\;$= 21.9, $\lambda_{4}\;$= 4.8. The twenty-dimensional test data were projected onto the first three eigenvectors (LDV1, LDV2 and LDV3) of LDA to generate three-dimensional data in Figure 4. Figure 4 illustrated the scattergram of data points performed by PCA + LDA. This figure showed that the data points of Guangming and Mengniu have crossed parts, which are difficult to be distinguished. It was clear that PCA + LDA has a certain classification function for data, but it could not clearly depict the classification boundary. Therefore, this experiment used the improved null linear discriminant analysis (iNLDA) to improve the data distribution for classification.

### 3.3. Discriminant Feature Extraction by iNLDA

**Figure 5:**
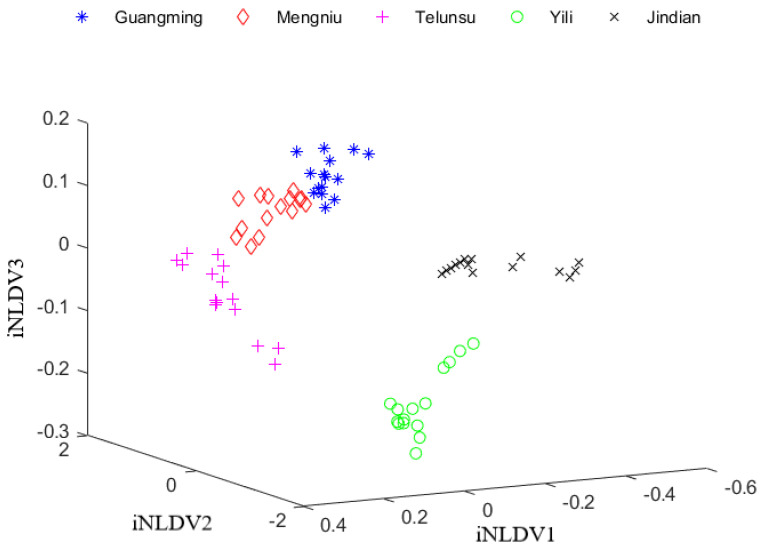
The data distribution of test set after PCA + iNLDA.

iNLDA can increase the compactness of data points in the same class and separation of data points in different classes. The class of data points can be accurately determined by classifiers, like the KNN classifier. After iNLDA processed the training data to generate four discriminant vectors (iNLDV1, iNLDV2, iNLDV3 and iNLDV4), the 20-dimensional test samples were transformed into the 4-dimensional data, whose first three-dimensional data were shown in Figure 5. Five kinds of milk samples are distributed clearly. However, the data distributions of Guangming, Mengniu and Telunsu were a little close. Therefore, there might still be problems such as misclassification and identification failure. Nevertheless, compared with LDA, the recognition accuracy of iNLDA has been greatly improved.

### 3.4. Discriminant Feature Extraction by FiNLDA

To reduce the limitation of iNLDA, FiNLDA was introduced to improve the recognition accuracy and avoid the misclassification. This experiment introduced fuzzy membership to initialize the training samples to improve the performance of processing overlapped data. The parameters of FiNLDA were set: the fuzzy index *m* = 2.0, the number of class *c* = 5. The fuzzy membership degrees were calculated by the equation described in fuzzy c-means (FCM) clustering. The initial cluster centers of FiLDA were the mean values of each brand of milk samples, and they are shown in Equation (6) with application of the initial cluster center model to classification and calculation accuracy.

**Figure 6:**
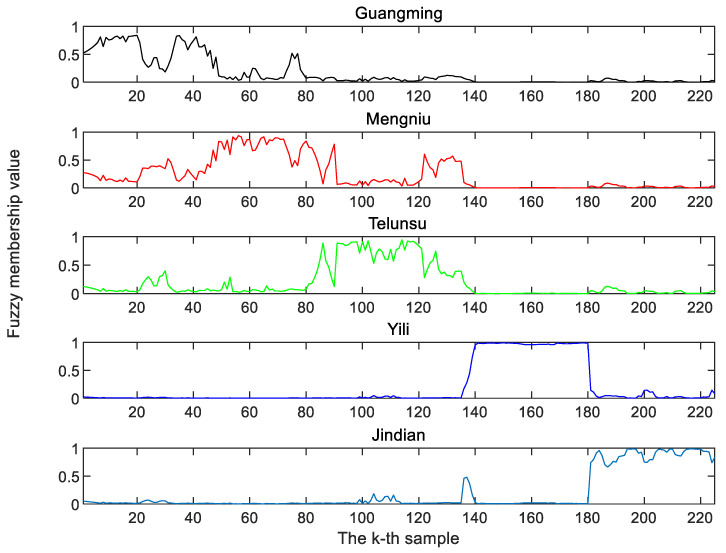
Fuzzy membership of FiNLDA.

The initial fuzzy membership values of FiNLDA are shown in Figure 6. The abscissa represents the sample data and the ordinate stands for the fuzzy membership value. This experiment involved five different brands of milk samples. Hence, there are five different little figures. Each little figure represents a brand of milk, i.e., Guangming, Mengniu, Telunsu, Yili and Jindian. When the fuzzy membership degree of the *i*-th sample u*_ij_* is the largest in the *j*-th class, it can be determined that the *i*-th sample belongs to the *j*-th class.

**Figure 7:**
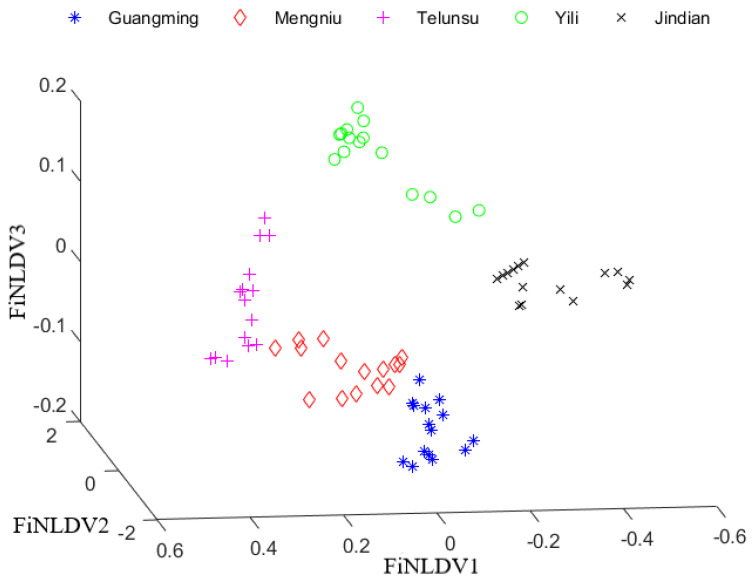
The data distribution after PCA + FiNLDA.

After PCA + FiNLDA, the data distribution is shown in Figure 7. From the fuzzy membership and data distribution, it could be seen that different brands of milk samples can be separated well by the FiNLDA algorithm.

(6) $$v^{\left(0\right)}=\left(\begin{array}{c} v_{1}^{\left(0\right)}\\ v_{2}^{\left(0\right)}\\ v_{3}^{\left(0\right)}\\ v_{4}^{\left(0\right)}\\ v_{5}^{\left(0\right)} \end{array}\right)={\left(\begin{array}{rrrrr} 1.0527 & -0.2374 & -0.0451 & \cdots & 0.0016\\ 0.8488 & 0.1277 & -0.0212 & \cdots & -0.0022\\ 0.3955 & 0.3278 & 0.0478 & \cdots & 0.0020\\ -1.7051 & 0.1550 & -0.0257 & \cdots & -0.0018\\ -0.6238 & -0.3968 & 0.0360 & \cdots & 0.0017 \end{array}\right)}_{5\times 20}$$

### 3.5. Classification Results

The K-nearest neighbor (KNN) classifier has many advantages. For example, it is simple and effective, and it is suitable for cross-domain samples and automatic classification of large samples. This experiment can use its characteristics to classify the milk samples. This study used KNN to compare the classification accuracies of different algorithms and different values of K. MATLAB was applied to calculate the accuracy of the KNN classifier. Take K = 3 for example. The experimental results illustrated that the identification accuracies of LDA, iNLDA and FiNLDA are 74.7%, 88% and 93.3%, respectively. It turned out that FiNLDA has a good classification effect on milk brands.

### 3.6. Classify Accuracy under Different Values of K

**Figure 8:**
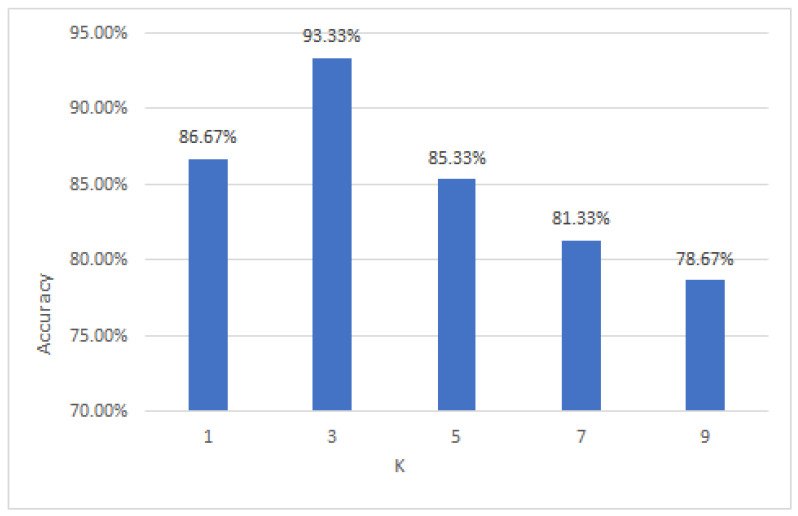
Classification accuracy under different K values of KNN.

It is well known that different values of K for a KNN classifier can affect the classification accuracy. If this experiment chooses a smaller K value, our model will become complex and easily overfitted. Additionally, the selection of a larger K value in our experiment represents prediction with the training data in a larger neighborhood, thus resulting in the wrong prediction since the training examples that are far away from the input examples (not similar) will also be involved in the prediction. An increase in the K value indicates that the model is simplified. Therefore, it is advisable to find the optimal K value. This study could use the different K values of the KNN classifier and compare the classification accuracy of the classifier. On this basis, this study calculated the classification accuracy under different K values to screen the optimal K value to achieve the classification goal. The classification results were shown in Figure 8. After the comparison, this experiment found when the K value reached to 3, the classification accuracy was the highest. Therefore, this experiment chose 3 as the K value of the KNN classifier.

## 4. Discussion

In order to identify the brand of milk samples correctly, this study aimed to extract discriminant features effectively from the NIR spectra of milk samples by proposing a novel fuzzy feature extraction method, i.e., FiNLDA, which was a combination of a fuzzy set and iNLDA. The NIR spectra of milk samples contained a noise signal and they were overlapped seriously, and this made it difficult to identify the spectra of different brands of milk samples. As the “hard” feature extraction method, LDA and iNLDA are not satisfactory in the results of processing spectral data. FiNLDA, as a “soft” feature extraction method, demonstrated the excellent ability to process spectral data. The experiment showed that FiNLDA achieved the highest classification accuracy of 93.33%, which was higher than LDA and iNLDA. Furthermore, the FiNLDA-based classification model was tested with different K values of KNN, and the classification accuracies were higher than 85% when K was 1, 3 and 5, respectively.

**Figure 9:**
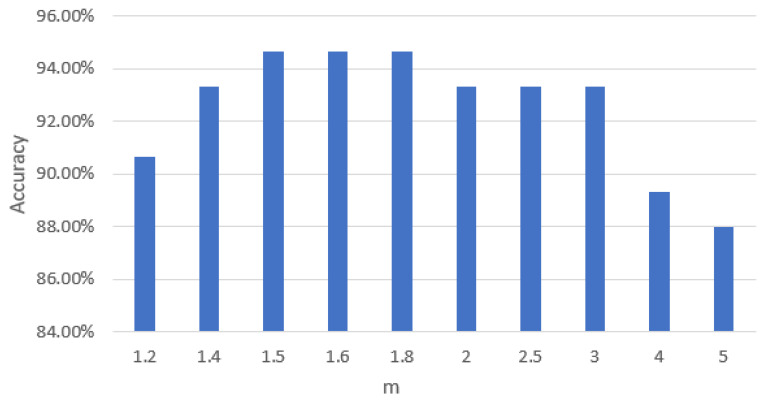
Classification accuracy under different weight indices.

As a fuzzy discriminant analysis, the FiNLDA algorithm used the fuzzy between/within scatter matrix to compute eigen decomposition, and the weight index *m* in the matrix has a powerful influence on the classification accuracy. If *m* becomes larger, the $u_{ij}^{m}$ is smaller and “fuzzier”. When m→+∞, the fuzzy membership $u_{ij}^{}\to 1/c$ [29], and usually $0\leq u_{ij}^{}<1$, thus, $u_{ij}^{m}\to 0$. On the other hand, when m→1, the fuzzy between/within scatter matrix becomes the “hard” between/within scatter matrix. The choice of a suitable m is still an open problem and has no theoretical basis. This study tried different *m* weight exponents to figure out the classification accuracies, which were shown in Figure 9. From Figure 9, the classification accuracy of FiNLDA reached the highest of 94.67% when the optimal value of *m* was 1.5, 1.6 and 1.8.

## 5. Conclusions

The NIR spectra of milk samples have the question of overlap and noisy data, which brings difficulty to classifying the spectra. To increase the identification accuracy, a new fuzzy feature extraction algorithm, i.e., FiNLDA, was proposed by combining fuzzy theory with iNLDA, and it extracted the discriminant features from the NIR spectra. To classify milk brands quickly, nondestructively and effectively, the method of uniting the FiNLDA algorithm with SG filtering and PCA was designed in this study. At first, near-infrared spectra of 300 milk samples from five brands were acquired by a NIR-M-R2-type near-infrared spectrometer, and they were preprocessed by a SG-filtering algorithm. Secondly, the spectra were compressed by PCA, and extracted by LDA, iNLDA and FiNLDA, respectively. Finally, KNN was performed to identify milk brands. Compared with LDA and iNLDA, FiNLDA can accurately identify milk brands and has the highest classification accuracy. On the basis of the accurate classification of milk brands by FiNLDA, this study confirmed the feasibility of identifying milk brands by combining the portable NIR spectrometer and FiNLDA. 

## Data Availability

The data used to support the findings of this study can be made available by the corresponding author upon request.
